# A distribution-centered approach for analyzing human adipocyte size estimates and their association with obesity-related traits and mitochondrial function

**DOI:** 10.1038/s41366-021-00883-6

**Published:** 2021-06-25

**Authors:** Julius Honecker, Dominik Weidlich, Simone Heisz, Cecilia M. Lindgren, Dimitrios C. Karampinos, Melina Claussnitzer, Hans Hauner

**Affiliations:** 1 Technical University of Munich, Else Kröner-Fresenius-Center for Nutritional Medicine, Chair of Nutritional Medicine, School of Life Sciences, Gregor-Mendel-Straße 2, 85354 Freising-Weihenstephan, Germany; 2 Department of Diagnostic and Interventional Radiology, School of Medicine, Technical University of Munich, Munich, Germany; 3 Big Data Institute at the Li Ka Shing Centre for Health Information and Discovery, University of Oxford, Oxford, UK; 4 Broad Institute of MIT and Harvard, Cambridge, MA USA; 5 Division of Gerontology, Department of Medicine, Beth Israel Deaconess Medical Center, Boston, MA USA; 6 Harvard Medical School, Harvard University, Boston, MA USA; 7 Institute for Nutritional Medicine, School of Medicine, Technical University of Munich, Georg-Brauchle-Ring 62, 80992 Munich, Germany

**Keywords:** Fat metabolism, Energy metabolism

## Abstract

**Objective:**

Cell diameter, area, and volume are established quantitative measures of adipocyte size. However, these different adipocyte sizing parameters have not yet been directly compared regarding their distributions. Therefore, the study aimed to investigate how these adipocyte size measures differ in their distribution and assessed their correlation with anthropometry and laboratory chemistry. In addition, we were interested to investigate the relationship between fat cell size and adipocyte mitochondrial respiratory chain capacity.

**Methods:**

Subcutaneous and visceral histology-based adipocyte size estimates from 188 individuals were analyzed by applying a panel of parameters to describe the underlying cell population. Histology-based adipocyte diameter distributions were compared with adipocyte diameter distributions from collagenase digestion. Associations of mean adipocyte size with body mass index (BMI), glucose, HbA_1C_, blood lipids as well as mature adipocyte mitochondrial respiration were investigated.

**Results:**

All adipocyte area estimates derived from adipose tissue histology were not normally distributed, but rather characterized by positive skewness. The shape of the size distribution depends on the adipocyte sizing parameter and on the method used to determine adipocyte size. Despite different distribution shapes histology-derived adipocyte area, diameter, volume, and surface area consistently showed positive correlations with BMI. Furthermore, associations between adipocyte sizing parameters and glucose, HbA_1C_, or HDL specifically in the visceral adipose depot were revealed. Increasing subcutaneous adipocyte diameter was negatively correlated with adipocyte mitochondrial respiration.

**Conclusions:**

Despite different underlying size distributions, the correlation with obesity-related traits was consistent across adipocyte sizing parameters. Decreased mitochondrial respiratory capacity with increasing subcutaneous adipocyte diameter could display a novel link between adipocyte hypertrophy and adipose tissue function.

## Introduction

Adipose tissue is a unique organ with great plasticity displaying unmatched expansion and shrinkage capacities during periods of caloric excess or deprivation which is directly reflected in up to multiple fold changes in fat cell volume [1–3]. The onset of obesity in adults is characterized by an increase in adipose tissue mass mostly due to the enlargement of existing fat cells (hypertrophy) [4, 5]. Hyperplasia, which describes an increase in fat cell number is considered to only play a minor role in the expansion of adipose tissue mass in adults [5].

To measure adipocyte size, three methods have emerged as standards: histological sections, collagenase digestion, and osmium tetroxide fixation. Despite systematic size differences, all methods are largely coherent regarding their size estimate association with obesity-related traits [6]. However, adipocyte size distributions from different adipocyte sizing methods vary in characteristic features of the distribution (i.e., modality and symmetry) [6]. Furthermore, distributions from one sizing method may not always be comparable since different variables such as area or feret diameter can be used as output measurements [7, 8]. In addition, the transformation of the initial size measurement into another sizing variable assuming a spherical shape might reduce comparability due to the nonlinear transformation of the data (diameter = sqrt((4*area)/*π*); volume = 1/6*diameter^3^*π) [8–10].

Adipocyte diameter and volume are usually related to anthropometry, metabolic outcomes, or adipocyte function [5, 10, 11]. Due to the introduction of fast and automated adipocyte size determination software, adipocyte area is currently reported more frequently [12, 13]. Adipocyte volume as a measure of fat cell size has high physiological relevance and is directly related to lipid storage capacity and adipose tissue cellularity [5, 6, 14, 15]. Furthermore, associations of fat cell size with comorbidities of obesity (Type 2 diabetes (T2D), dyslipidemia, and cardiovascular disease) have been reported and insulin-resistant individuals were found to exhibit larger visceral adipocytes compared to insulin-sensitive controls [16–20]. In addition, studies on size-separated fat cells provide evidence for functional differences between small and large adipocytes from the same individual [10, 21, 22]. However, cross-sectional studies assessing the genetic and metabolic underpinnings of adipocyte hypertrophy remain scarce [12, 14, 23–25]. Despite their low content in comparison with other tissues, adipocyte mitochondria are essential organelles for adipocyte differentiation, lipogenesis, adipokine secretion, and browning [26–29]. Emerging evidence indicates a fundamental relationship between obesity and altered adipocyte mitochondrial metabolism [30–32]. Considering the central role of mitochondria in adipose tissue metabolism and their association with obesity-related traits it seems likely that adipose tissue mitochondrial function could be associated with fat cell size. However, the relationship between adipocyte size and mitochondrial function has not yet been investigated cross-sectionally in humans, but solely in size-separated adipocytes [33, 34].

Therefore, this study aimed to achieve a comprehensive overview of similarities and differences in fat cell size distribution shape dependent on the adipocyte sizing method and the extracted sizing parameter. Furthermore, we sought to relate measurements of adipocyte size to phenotypic and laboratory variables. We used mature adipocyte respirometry data to investigate if the mitochondrial function is associated with adipocyte size.

## Materials and methods

### Study participants and phenotype data

Sc and vc adipose tissue samples were obtained from 188 adult individuals (129 female, 59 male) undergoing elective abdominal surgery. Each participant gave written informed consent before inclusion and the study protocol was approved by the ethics committee of the Technical University of Munich (Study No. 5716/13). Collected data included the presence or absence of T2D as diagnosed by the treating physician, demographics (age and sex), anthropometry (weight before surgery, height, and BMI) as well as laboratory values (glycated HbA_1C_, fasting plasma glucose, triglycerides, cholesterol, LDL, and HDL).

Study participants were on average 48 ± 13 years old with a mean BMI of 43.6 ± 13.3 kg/m^2^. According to BMI categories 23 individuals with normal weight (12.2%), 18 individuals with overweight (9.6%), 8 individuals with obesity class I (4.3%), 13 individuals with obesity class II (6.9%), and 122 individuals with obesity class III (64.9%) participated in the study.

T2D was diagnosed in 45 individuals (24.3%), while 140 participants were free of T2D (3 not known). The study populations’ characteristics are summarized in Table 1 and Table S1.

**Table 1:** Study participants’ characteristics.

Variable	Mean ± SD	Range (min–max)
*Anthropometry*		
Age [years]; *n* = 188	48 ± 13	18–78
BMI [kg/m^2^]; *n* = 184	43.6 ± 13.3	18.2–83.3
*Glucose homeostasis*		
Glucose [mmol/l]; *n* = 139	6.0 ± 3.0	2.2–22.1
HbA_1C_ [%]; *n* = 99	6.0 ± 1.2	4.6–11.5
*Lipids*		
Total cholesterol [mmol/l] *n* = 97	5.1 ± 1.0	1.4–8.0
LDL [mmol/l]; *n* = 92	3.1 ± 0.8	1.3–5.3
HDL [mmol/l]; *n* = 94	1.3 ± 0.4	0.6–2.4
Triglycerides [mmol/l]; *n* = 96	1.9 ± 1.0	0.6–6.8

### Adipose tissue sampling and adipocyte isolation

Sc (*n* = 161) and vc (*n* = 188) adipose tissue biosamples were obtained from the upper abdominal area at the site of incision and in the proximity of the angle of his, respectively. Paired sc and vc adipose tissue were available from 146 individuals. After excision, samples were immediately fixed in 4% formaldehyde for later use in histology. Tissue samples for mature adipocyte isolation were transported to the laboratory in DMEM-F12 (Thermo Fisher Scientific, Waltham, Massachusetts) + 1% penicillin–streptomycin (Merck, Darmstadt, Germany) where collagenase-based mature adipocyte isolation was carried out as described previously [33].

### Diameter determination of floating mature adipocytes

Approximately, 50 μl of the adipocyte suspension was pipetted onto a glass slide and the diameter of 100 cells was manually determined under a light microscope. Adipocyte diameter from collagenase digestion was available for 84 sc and 97 vc samples.

### Histology-based adipocyte cross-sectional area determination

At least three 5 µm thick hematoxylin and eosin-stained sections with a minimum distance of 100 µm or from different blocks were used for microscopy and size determination. The adipocyte size estimates used for analysis were assessed retrospectively based on a recently published method, the Adipocyte U-Net [12]. Objects with an area smaller than 200 µm^2^ and larger than 16,000 µm^2^ were excluded as they typically represent artifacts from tissue processing and histology.

### Adipocyte area parameters

Totally, 500 cell cross-sectional area estimates and 100 cell diameter estimates were used for data analysis on histology and collagenase-digested adipocytes, respectively. If not given by the initial measurement adipocyte area, diameter, volume or surface area were calculated based on the assumption of spherical shape. Descriptive statistics (density plots and quantile-quantile plots), as well as measures for central tendency (arithmetic mean and median), were calculated. In addition, the first and ninth decile were determined and the interdecile range (IDR) was calculated as a measure of the dispersion of adipocyte sizes. Skewness was used as a distribution asymmetry measure and kurtosis was used to assess distribution tailedness.

### Respirometry

Data on mature adipocyte mitochondrial function was available for 24 sc and 35 vc samples originating from an earlier project of our group [30]. Briefly, 200 µl of packed isolated adipocytes were pipetted into the experimental chamber of an Oxygraph-2k (Oroboros Instruments, Innsbruck, Austria) and substrate-uncoupler-inhibitor titration was carried out to assess the following respiratory states: (I) Free OXPHOS capacity which describes the respiratory capacity potentially available for ADP phosphorylation, (II) OXPHOS capacity describing the respiratory capacity of mitochondria in the ADP activated state, (III) electron transfer system capacity (ETS) describing the maximum respiratory chain capacity due to chemical uncoupling introducing proton reflux into the mitochondrial matrix, and (IV) leak respiration in the presence of oligomycin describing the dissipative component of respiration that does not contribute to ATP synthesis [35]. DNA from adipocytes was isolated for normalization purposes and results were given as picomoles of oxygen consumed per second and nanogram of DNA. Respiration was measured in mature adipocytes since cell-mitochondrion interactions are preserved [36]. In addition, isolated adipocytes offer the advantage that confounding effects from the stromal vascular fraction on respiration and normalization can be ruled out [37].

### Statistics

Shapiro Wilk tests and quantile-quantile plots were used to test for/against normality. All data were presented as mean ± SD. If not stated otherwise, Pearson correlation was used to test for associations between adipocyte size, anthropometry, laboratory values, and mitochondrial respiration. To further explore the relationship between adipocyte size and phenotype data multiple linear regression was used to adjust for differences in BMI between individuals. *P* values below 0.05 were considered significant. All analyses were conducted in *R* [38].

## Results

### Histology-based calculation of adipocyte diameter or volume from adipocyte area changes the adipocyte size distribution shape

**Fig. 1:**
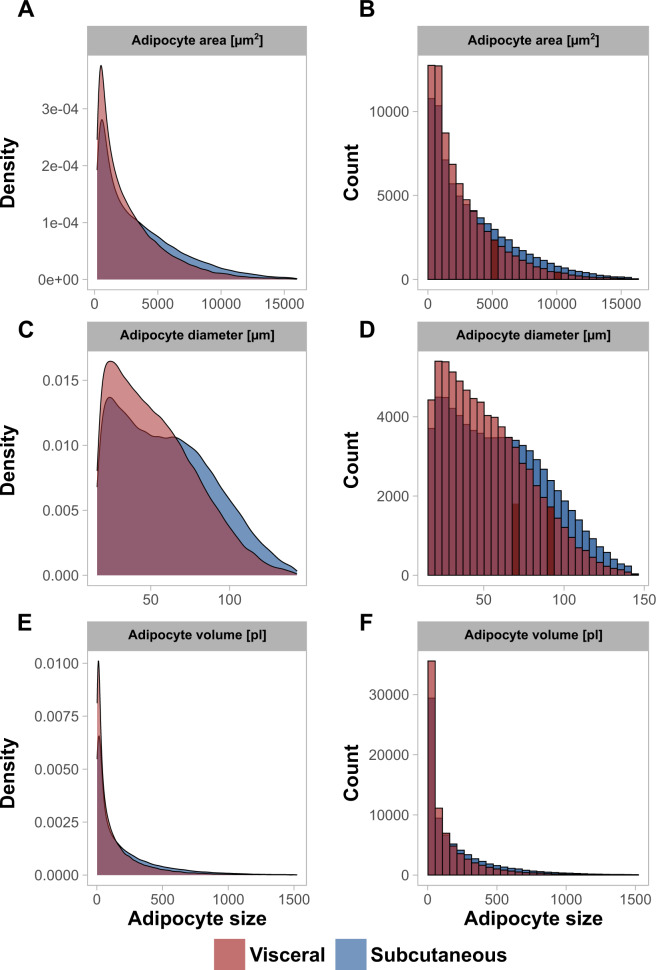
Histology-derived adipocyte size distributions for adipocyte area, diameter, and volume. Density plots (left) and histograms (right) are shown for adipocyte area (**A**, **B**), adipocyte diameter (**C**, **D**), and adipocyte volume (**E**, **F**). Density plots and histograms were calculated for the whole cohort, separated on adipose tissue depot (sc *n* = 161, vc *n* = 188) and each individual is equally represented with 500 adipocyte size estimates. Independent of the size parameter assessed all distributions are showing positive skewness. It is, however, visible that the calculation of adipocyte diameter from the area which represents a square root transformation (*d* = sqrt((4*A)/*π*)), broadens the distribution shape and results in less positive skewness. Calculation of volume from adipocyte area (*V* = 1/6*d^3^*π) results in a sharper distribution of values with increased positive skewness in comparison to the adipocyte area distribution.

The adipocyte area distributions across all individuals (Fig. 1A, B) did not fit a normal distribution and consistently showed positive skewness for both adipose depots (Table 2). This observation based on pooled data was in agreement with the subject and depot-specific distributions, consistently showing asymmetry as measured by positive skewness values (data not shown). *P* values < 0.05 from the Shapiro Wilk tests and large deviations in the correlation between a normal distribution and sample distribution displayed by quantile–quantile plots further supported the hypothesis of histology-derived adipocyte areas being non-normally distributed. Density plots, histograms, and quantile–quantile plots for each individual and depot are provided in Supplemental Figs. S1–S3. Due to the positive skew mean and median adipocyte area was not overlapping with the maximum of the distribution (mode) and therefore did not reflect the major fraction of the adipocyte population (Table 2). The calculation of adipocyte diameter from adipocyte area, which represents a non-linear square root transformation resulted in a broader distribution of the data (Fig. 1C, D). This observation was verified by decreased adipocyte diameter distribution skewness and kurtosis in comparison to the adipocyte area distributions (skew_sc area_ = 1.1 ± 0.4, skew_sc diameter_ = 0.3 ± 0.2; skew_vc area_ = 1.3 ± 0.4, skew_vc diameter_ = 0.4 ± 0.2; Table 2). Calculation of adipocyte volume from adipocyte area represents exponentiation of the data by the power of 3 and resulted in a sharper distribution shape with greater asymmetry compared to the adipocyte area distributions (Fig. 1E, F). In agreement, skewness and kurtosis of the adipocyte volume distribution increased in comparison to the adipocyte area distribution (skew_sc area_ = 1.1 ± 0.4, skew_sc volume_ = 1.9 ± 0.6; skew_vc area_ = 1.3 ± 0.4, skew_vc volume_ = 2.3 ± 0.8; Table 2). Since the surface area of a sphere equals four times the largest cross-sectional area (*A*_surface_ = 4π*r*^2^, *A*_Great circle_ = π*r*^2^, therefore *A*_surface_ = 4*A_Great circle_), calculation of adipocyte surface area from adipocyte cross-sectional area does neither change distribution shape, nor statistical outcome measures.

**Table 2:** Adipocyte size summary.

Histology	Depot	Mean	Median	First Decile	Ninth decile	IDR	Skewness	Kurtosis
*Histology*								
Area [µm^2^]	sc (*n* = 161)	3472.0 ± 886.8	2678.8 ± 795.4	443.2 ± 76.7	7726.3 ± 2069.0	7283.1 ± 2030.1	1.1 ± 0.4	4.0 ± 1.7
	vc (*n* = 188)	2748.8 ± 863.6	2099.8 ± 718.7	407.7 ± 68.1	6043.0 ± 2049.8	5635.3 ± 2013.6	1.3 ± 0.4	5.1 ± 2.5
Diameter [µm]	sc (*n* = 161)	59.6 ± 7.8	57.7 ± 8.8	23.7 ± 2.0	98.2 ± 13.8	74.6 ± 12.8	0.3 ± 0.2	2.3 ± 0.4
	vc (*n* = 188)	53.1 ± 8.4	50.9 ± 8.9	22.7 ± 1.9	86.3 ± 15.8	63.6 ± 14.8	0.4 ± 0.2	2.6 ± 0.6
Volume [pl]	sc (*n* = 161)	198.1 ± 73.2	107.7 ± 47.4	7.1 ± 1.9	524.7 ± 204.1	517.6 ± 203.1	1.9 ± 0.6	7.8 ± 5.1
	vc (*n* = 188)	141.5 ± 64.1	75.5 ± 38.3	6.3 ± 1.6	369.0 ± 176.9	362.7 ± 176.0	2.3 ± 0.8	11.0 ± 7.8
Surface area [µm^2^]	sc (*n* = 161)	13887.9 ± 3547.0	10715.0 ± 3181.5	1772.6 ± 8276.0	30905 ± 8267.0	29132.5 ± 8120.2	1.1 ± 0.4	4.0 ± 1.7
	vc (*n* = 188)	10,995 ± 3454.4	8399.2 ± 2875.0	1630.9 ± 272.5	24172.1 ± 8199.2	22541.2 ± 8054.3	1.3 ± 0.4	5.1 ± 2.5
*Collagenase digestion*								
Area [µm^2^]	sc (*n* = 84)	9523.7 ± 2375.1	9356 ± 2659.6	4259.9 ± 2307.4	14775.4 ± 3042.1	10515.5 ± 2784.8	0.6 ± 1.3	5.4 ± 10.7
	vc (*n* = 97)	8292.5 ± 2363.5	7949 ± 2507.2	3661.0 ± 1870.6	12955.0 ± 3384.7	9294.0 ± 2819.7	0.9 ± 1.3	6.3 ± 9.3
Diameter [µm]	sc (*n* = 84)	105.6 ± 14.7	107.9 ± 16.3	70.5 ± 21.3	136.4 ± 14.5	65.9 ± 20.0	−0.2 ± 0.9	3.9 ± 5.3
	vc (*n* = 97)	98.3 ± 15.3	99.2 ± 16.8	66.0 ± 17.4	127.2 ± 17.7	61.2 ± 16.9	0.1 ± 0.8	4.1 ± 4.0
Volume [pl]	sc (*n* = 84)	778.0 ± 273.9	701.8 ± 286.4	232.4 ± 173.8	1372.6 ± 412.9	1140.3 ± 352.0	1.3 ± 1.7	8.1 ± 15.1
	vc (*n* = 97)	639 ± 258.7	553.4 ± 248.6	182.4 ± 137.9	1138.1 ± 427.2	955.6 ± 367.0	1.8 ± 1.6	10.2 ± 13.6
Surface area [µm^2^]	sc (*n* = 84)	38094.8 ± 9500.3	37427.7 ± 10638.4	17039.5 ± 9229.8	59101.6 ± 12168.5	42062.1 ± 11139.3	0.6 ± 1.3	5.4 ± 10.7
	vc (*n* = 97)	33170.2 ± 9453.9	31796.8 ± 10028.7	14644.1 ± 7482.5	51820.1 ± 13538.7	37176.0 ± 11278.7	0.9 ± 1.3	6.3 ± 9.3

### Comparison between histological and collagenase-based adipocyte diameter distributions

**Fig. 2:**
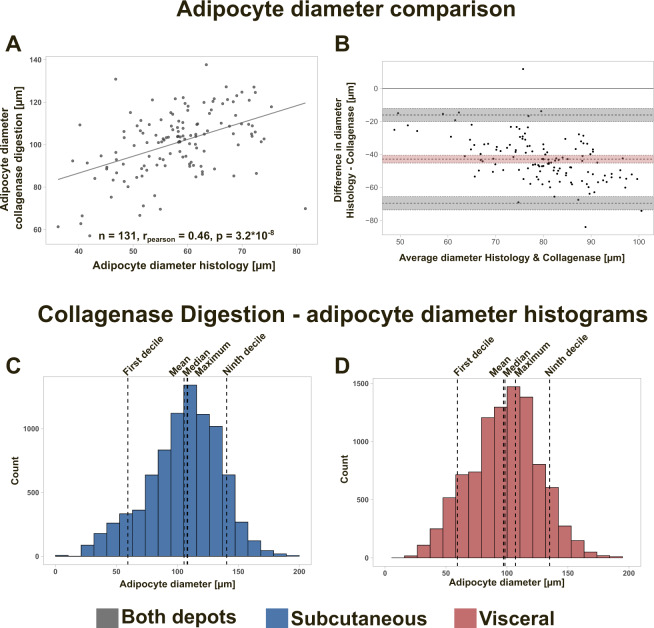
Comparison of mean adipocyte diameter between histology and collagenase digestion and collagenase-based adipocyte diameter distributions. A scatterplot (**A**) as well as a Bland–Altman plot (**B**) are shown for comparing mean adipocyte diameter from histology and collagenase digestion. For method comparison (**A**, **B**) sc and vc samples were pooled (*n*_tot_ = 131, *n*_sc_ = 56, *n*_vc_ = 75). Histograms were plotted for sc (**C**, blue) and visceral adipose tissue (**D**, red) to visualize adipocyte diameter distributions from collagenase digestion. Each individual is equally represented with 100 adipocyte size estimates from a total of 84 individuals (sc) and 97 individuals (vc). Dotted vertical lines show where the first decile, ninth decile, mean, median, and modal adipocyte diameter are located in the histogram. Mean adipocyte diameter from histology and collagenase digestion are concordant as indicated by Pearson-correlation (*r*_Pearson_ = 0.46, *p* = 3.2 × 10^−8^). However, as shown by the Bland–Altman plot (**B**) mean adipocyte diameter from histology was consistently smaller than mean adipocyte diameter from collagenase digestion. Independent of the depot, diameter histograms from collagenase digestion were following the pattern of a normal distribution (**C**, **D**).

Overall, mean adipocyte diameter derived from histology and collagenase digestion showed a good correlation with each other (*r*_Pearson_ = 0.46, *p* = 3.2 × 10^−8^; Fig. 2A). The mean adipocyte diameter from histology was, however, consistently smaller than the mean collagenase adipocyte diameter as shown by the Bland–Altman method comparison plotting (Table 2, Fig. 2B). This can be attributed to the fact that the adipocyte diameter from histology might not always be equivalent to the maximal diameter of the cell in vivo [8]. With collagenase digestion, mature adipocytes are liberated from their connective tissue and take on a spherical shape allowing to measure the maximal cell diameter under the microscope. Adipocyte diameter histograms indicate that the diameter distribution from collagenase digestion did fit the shape of a normal distribution (Fig. 2C, D) and was considerably less skewed in comparison to adipocyte diameter distributions from histology (Table 2).

### Mean adipocyte size measurements from histology show similar correlations with anthropometric variables and laboratory values

**Fig. 3:**
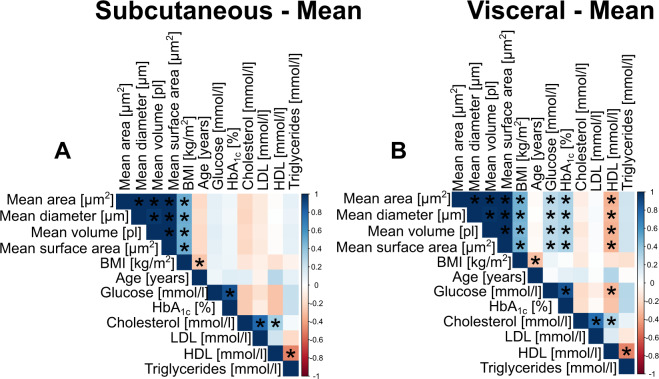
Correlations between mean adipocyte size, anthropometric variables, and laboratory values. Data are shown as a Pearson correlation matrix. Positive correlations are depicted in shades of blue while negative correlations are shown in shades of red. Asterisks indicate significant associations. *P* values were Bonferroni corrected to account for multiple testing. In the sc depot (**A**), only BMI showed significant associations with measures of mean adipocyte size. In the vc depot **(B)** BMI, glucose and HbA_1C_ showed positive associations with measures of mean adipocyte size. HDL was negatively associated with vc mean adipocyte size.

As previously published, measures of mean adipocyte size showed strong correlations with BMI in both adipose tissue depots (Fig. 3, Table S2). Significance levels and Pearson correlation coefficients were in a comparable range independent of the adipocyte sizing parameter that was used (*r*_Pearson_ = 0.46–0.49, *p* = 9 × 10^−12^–1 × 10^−10^). Other than BMI no significant associations with mean adipocyte size were found in sc fat. In addition to BMI, mean adipocyte size correlated with glucose (mean diameter: *n* = 139, *r*_Pearson_ = 0.26, *p* = 0.002), HbA_1C_ (mean diameter: *n* = 99, *r*_Pearson_ = 0.31, *p* = 0.002) and HDL (mean diameter: *n* = 94, *r*_Pearson_ = − 0.32, *p* = 0.002) in the vc fat depot (Fig. 3, Table S2). Notably, despite different individual distribution shapes, mean adipocyte cross-sectional area, diameter, volume, and surface area were all similarly correlated with BMI and laboratory values (Fig. 3, Table S2). Since our data and results from previous studies show that BMI is strongly correlated with adipocyte size multiple linear regression was used to rule out any possible effect of differences in BMI on the association of laboratory values with mean adipocyte diameter (Table S3). Results from our multiple linear regression analysis were in agreement with results from the correlation analysis. Mean sc adipocyte diameter was only associated with BMI, while mean vc adipocyte diameter was significantly associated with age, fasting plasma glucose, HbA_1C_, HDL and triglycerides (*p* < 0.05, Table S3). Besides the continuous variables that were considered in the correlation analysis sex and the absence or presence of T2D were added as categorical variables to our multiple linear regression model. The mean visceral adipocyte diameter of males was significantly larger compared to females in our multiple linear regression model (*p* = 0.006, Table S3). In agreement with the positive correlations of mean visceral adipocyte diameter with glucose and HbA_1C_, respectively, adipocytes of individuals with T2D were larger compared to the control group in a multiple linear regression model (*p* = 0.01, Table S3). Together, the observed changes in adipocyte distribution shape did not influence the correlation of extracted mean values with obesity-related traits.

### Associations of adipocyte size and mitochondrial function

**Fig. 4:**
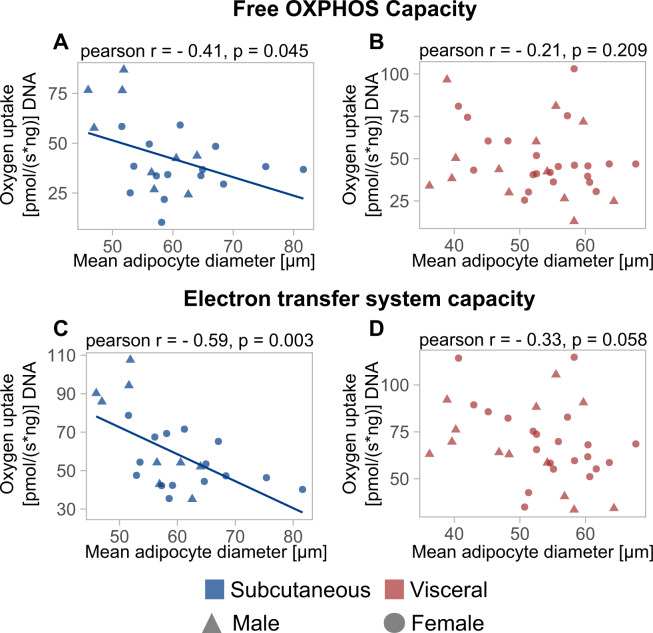
Correlations between mean adipocyte diameter and mitochondrial respiratory capacity of mature adipocytes. Data are shown in form of scatterplots. **A**, **B** The correlation between free OXPHOS capacity and mean adipocyte diameter for sc and vc adipocytes respectively. **C**, **D** The correlations between mean adipocyte diameter and electron transfer system capacity. Significant correlations between free OXPHOS capacity (*r*_Pearson_ = −0.41, *p* = 0.045) and electron transfer system capacity (*r*_Pearson_ = −0.59, *p* = 0.003) were observed for sc adipose tissue (**A**, **C**).

Since all mean adipocyte size parameters showed comparable correlations with anthropometry and laboratory chemistry, the relationship between adipocyte size and mitochondrial respiratory capacity was only investigated using mean adipocyte diameter. Correlation analysis showed an inverse relationship between sc free OXPHOS capacity and mean adipocyte diameter (Fig. 4, *n* = 24, *r*_Pearson_ = −0.41, *p* = 0.045). In addition, significant correlations between mean adipocyte diameter and OXPHOS capacity (*n* = 24, *r*_Pearson_ = −0.53, *p* = 0.008) as well as ETS capacity (*n* = 24, *r*_Pearson_ = −0.59, *p* = 0.003) were observed (Fig. 4, Table S4). Both sc and vc mean adipocyte diameter showed negative correlations with leak respiration in the presence of oligomycin (Table S4). We observed a weaker though significant association of OXPHOS capacity with mean adipocyte diameter in the vc depot (*n* = 35, *r*_Pearson_ = −0.36, *p* = 0.035) (Table S4). When adjusting for the effect of BMI on the association between mitochondrial respiration and mean adipocyte diameter, we did not observe a significant effect (Table S5). Initially, 500 sampled cells per depot and individual were used to obtain individual histology-derived adipocyte size distributions. This, however, resulted in the exclusion of study participants where only a little adipose tissue was available for histology. In a second analysis, we, therefore, reduced the number of sampled cells per individual and depot to 200, thereby increasing paired adipocyte size and respirometry data (*n*_sc_ = 32, *n*_vc_ = 41). Mean adipocyte diameters from 500 and 200 sampled cells were highly correlated (*r*_Pearson_ = 0.97, *p* < 2.2 × 10^−16^, Fig. S4). Sc and vc mean adipocyte diameter from 200 cells was correlated with OXPHOS capacity, leak respiration, and ETS capacity while free OXPHOS capacity was solely associated with sc mean adipocyte diameter (Table S6, Fig. S5). Significant associations between OXPHOS capacity (*ß* = −0.16, *p* = 0.007), leak respiration (*ß* = −0.29, *p* = 0.011) and ETS capacity (*ß* = −0.221, *p* = 0.001) with mean adipocyte diameter from 200 cells were present for the sc depot after adjusting for the effect of BMI in a multiple linear regression model (Table S7). No significant associations between mean adipocyte diameter and respiratory chain function except for ETS capacity (*ß* = −0.14, *p* = 0.045) were found when applying the multiple linear regression model to the vc depot (Table S7). In conclusion, adipocyte respiratory capacity was predominantly associated with mean adipocyte diameter in the sc depot.

## Discussion

We applied a series of analytical approaches to histology and collagenase-derived adipocyte size estimates with the goal to obtain an in-depth characterization of the underlying adipocyte size distributions and compare their relationship with phenotype or laboratory chemistry. Since mitochondrial dysfunction is related to obesity and/or T2D we investigated whether mitochondrial respiration is associated with adipocyte hypertrophy.

Our data indicate that histology-derived adipocyte cross-sectional area is characterized by positive skewness. Similar distribution shapes have been previously reported in rodents and humans [8, 39, 40]. We show that calculation of adipocyte diameter, volume or surface area from cross-sectional area introduces substantial changes to distribution shape. These changes are caused by mathematical data transformation and are therefore not only limited to human adipose tissue sections but apply to all species with similar underlying size distributions. In agreement with previous studies, adipocyte diameter distributions from collagenase digestion show greater symmetry, follow the shape of a normal distribution and the mean collagenase diameter is consistently larger than the mean histology diameter [6, 7, 41]. With isolated adipocytes, both Gaussian distributions and bimodal distributions containing a second smaller cell population are reported [6, 21, 42, 43]. Our study cannot confirm the presence of the second peak in adipocyte size distributions from isolated adipocytes and therefore supports the notion that size distributions from isolated adipocytes are unimodal and normally distributed. Density histograms from our study suggest that adipocyte size distributions from histology are unimodal but differ from isolated adipocytes by showing considerable skew (Figs. S1–S3). Despite 500 counted cells being sufficient to extract mean adipocyte size estimates it should be acknowledged that higher cell counts would in our opinion be desirable to reliably detect (if present) additional modes at lower frequencies [40, 44]. Using whole slide scans could display one method to achieve greater counts and therefore the question of modality should be addressed in future studies [12]. While the mean collagenase-derived adipocyte diameter represents the most frequent adipocyte population this is not the case for histology-derived distributions due to positive skew. Therefore, alternative measurements of adipocyte size such as IDR or modal adipocyte size are proposed to better describe non-normal distributions.

Besides different distribution shapes, we demonstrate that adipocyte area, diameter, volume, and surface area are equally correlated with common anthropometry and laboratory values. Across different sizing parameters, the correlation between adipocyte size and measures of glucose homeostasis and blood lipids was limited to the vc depot. Similar results were observed in previous studies and our results are in agreement with the well-known association between visceral obesity and metabolic disorders [11, 45]. Therefore, non-linear data transformation of the initial output variable (area) assuming spherical shape does not influence the associations between adipocyte size and obesity-related traits. Similar to inter-method comparisons we conclude that transformed adipocyte sizing parameters from the same method are equally related to measures of metabolic health and obesity [6]. Our data suggests that while the “ground truth” adipocyte size remains unknown and shows variation between studies reported associations with adipocyte size should in general be comparable. In our opinion, small cohort sizes and limited adipocyte counts propose greater limitations to significance and comparability rather than the utilization of different measurement methods or sizing parameters.

Mean adipocyte diameter, volume, and area are the most common adipocyte size measures [6, 11–13]. Since mature adipocytes are three-dimensional in shape and mainly comprised of a triglyceride-containing unilocular lipid droplet, volume is of high biological relevance for many research questions including fat mass and cellularity calculations [5, 46, 47]. Less biological relevance can be attributed to adipocyte area or diameter as they do not directly resemble the physiological properties of the adipocyte. Surface area is reported less frequently and is directly proportional to the largest cross-sectional area when the spherical shape is assumed. Since the surface area is proportional to membrane area it is relevant for receptor binding and signaling. Due to its linear relationship with adipocyte surface area, cross-sectional area could also be used as a meaningful readout of adipocyte size when signaling or biochemical processes are investigated. It is, however, important to emphasize that the cross-sectional area measured in histology is not necessarily equal to the largest cross-sectional area of the cell.

Considering the inherent advantages and limitations of each adipocyte sizing method as well as the different underlying distributions our study emphasizes that adipocyte size cannot be generalized, but always needs to be seen in context and interpreted regarding physiological relevance.

As a novel finding, our study correlates mean adipocyte diameter with mitochondrial function and our data indicate that individuals with larger sc adipocytes show decreased mature adipocyte respiratory capacity. To the best of our knowledge, there are no previous studies investigating adipocyte size and mitochondrial respiration and data is solely available for size-separated adipocytes where no differences in mitochondrial function between large and small adipocytes from the same individual were observed [33, 34]. Therefore, it can be speculated that individuals with hypertrophic adipocytes exhibit an overall decreased mitochondrial respiratory capacity independent of intrinsic adipocyte size variation. Our findings regarding the association of mean adipocyte diameter and respiratory chain function are in line with data suggesting that adipocyte mitochondrial function is altered in individuals with obesity [30, 48].

If mitochondria of hypertrophic adipocytes cannot provide sufficient ATP for cellular processes and lipid metabolism due to e.g., defects in the respiratory chain it is likely that this could manifest for example in elevated free fatty acid levels and increased production of reactive oxygen species (ROS) both promoting a pro-inflammatory environment. Independent of adipocyte size we could not detect differences in respiratory states between depots except for leak respiration in the presence of oligomycin (*p* = 0.003, Figure S6). Proton leak is thought to be the predominant component of leak respiration and has been identified as an important mechanism to protect against ROS [49]. As ROS are able to induce proton leak thus suggesting a feedback loop, elevated leak respiration in vc adipocytes could also resemble a cytoprotective mechanism against increased oxidative stress. Decreased leak respiration with larger adipocyte size and thus less protection against ROS could provide an explanation for the pro-inflammatory profile of hypertrophic adipocytes. Both hypotheses would be in line with literature reporting that vc adipose tissue and hypertrophic adipocytes are more prone to provide a pro-inflammatory environment compared to their sc and hyperplasic counterparts [10, 11, 50]. Additional studies that directly measure mitochondrial superoxide production and inducible uncoupling are however necessary to clearly elucidate the amount of ROS production in dependency of fat cell size and depot.

As respiration was shown to be cell type-specific differences in respiration and its relationship with cell diameter could also originate from the fact that sc and vc adipocytes arise from different precursors [51, 52]. Transcriptome profiling studies from multiple different adipose tissue sites further support this observation as sc and vc adipose tissues are clearly separated by gene expression [53]. Since vc adipose tissue was sampled in the proximity of the angle of his we cannot rule out that our results are specific to this compartment. Intra-visceral depot comparisons however suggest that different vc adipose tissue depots show similarity regarding their gene expression [53]. Thus, comparability between our biopsy site with other vc sites should be given. Intra-depot comparisons from the same subject should however be the objective of future studies to identify similarities and differences in metabolic function and to further stratify the current adipose tissue classification.

In conclusion, the results of the present study highlight the importance of ascertaining method, sizing parameter, and distribution when analyzing adipocyte size. The data suggest that mean and median adipocyte area can deviate substantially from the most frequent cell population if the underlying distribution is non-normal. Additional parameters are, therefore, proposed to complement previous adipocyte sizing methods to enable a more in-depth description of histology-derived adipocyte size towards a distribution-centered approach. Despite different distribution shapes histology-based adipocyte area, diameter, volume, and surface area are all equally related to clinical variables that have been frequently associated with adipocyte size. The association between sc mean adipocyte diameter and mitochondrial respiration represents a new finding of how adipose tissue metabolism is linked to adipocyte hypertrophy.

## Supplementary information

Supplementary Material
