# Supplementary material for: A distribution-centered approach for analyzing human adipocyte size estimates and their association with obesity-related traits and mitochondrial function
Source: Int J Obes (Lond). 2021 Jun 25;45(9):2108–17. doi: 10.1038/s41366-021-00883-6 (PMC8380540; doi:10.1038/s41366-021-00883-6)
Supplement: Supplementary file 1 — Supplementary Material [file 41366_2021_883_MOESM1_ESM.pdf]

SUPPLEMENTARY INFORMATION

A distribution-centered approach for analyzing human adipocyte size estimates and their association with obesity-related traits and mitochondrial function.

AUTHORS

Julius Honecker[1*], Dominik Weidlich[2], Simone Heisz[1], Cecilia M. Lindgren[3,4], Dimitrios C. Karampinos[2], Melina Claussnitzer[4,5,6], Hans Hauner[1,7$],

AFFILIATIONS

[1] Technical University of Munich, Else Kröner-Fresenius-Center for Nutritional Medicine, Chair of Nutritional Medicine, School of Life Sciences, Gregor-Mendel-Straße 2, 85354 Freising-Weihenstephan, Germany

[2] Department of Diagnostic and Interventional Radiology, School of Medicine, Technical University of Munich, Munich, Germany

[3] Big Data Institute at the Li Ka Shing Centre for Health Information and Discovery, University of Oxford

[4] Broad Institute of MIT and Harvard, Cambridge, MA, USA

[5] Division of Gerontology, Department of Medicine, Beth Israel Deaconess Medical Center, Boston, MA, USA

[6] Harvard Medical School, Harvard University, Boston, MA, USA

[7] Institute for Nutritional Medicine, School of Medicine, Technical University of Munich, Georg-Brauchle-Ring 62, 80992 München

RUNNING TITLE:

Adipocyte size distributions and their associations with obesity and mitochondrial function

KEYWORDS:

adipocyte size, adipocyte distribution, obesity, hypertrophy, adipose tissue, mitochondrial function

[$] CONTACT INFO

Prof. Hans Hauner

Technical University of Munich

Else Kröner-Fresenius-Center of Nutritional Medicine (EKFZ)

Gregor-Mendel-Str. 2

85354 Freising-Weihenstephan, Germany

Tel: +49-8161-712001;

Fax: +49-8161-712097

Mail: hans.hauner@tum.de

Ground mail address: nutritional.medicine@wzw.tum.de

Table S1: Study participants' characteristics according to BMI categories.

Variable	All Samples<br>n = 188	Normal weight<br>n = 23	Overweight<br>n = 18	Obesity class I<br>n = 8	Obesity class II<br>n = 13	Obesity class III<br>n = 122
**Sex**						
Female	129 (69%)	8 (35%)	6 (33%)	6 (75%)	10 (77%)	96 (79%)
Male	59 (31%)	15 (65%)	12 (67%)	2 (25%)	3 (23%)	26 (21%)
**Age**<br>**[years]**	48 ± 13<br>18 - 78	51 ± 17<br>22 - 78	61 ± 11<br>32 - 77	53 ± 8<br>42 - 64	47 ± 13<br>28 - 74	46 ± 11.<br>18 - 73
**BMI**<br>**[kg/m^2]**	43.6 ± 13.3<br>18.2 - 83.3	22.6 ± 2.1<br>18.2 - 24.9	27.1 ± 1.2<br>25.0 - 29.9	32.6 ± 1.5<br>30.5 - 34.7	37.4 ± 1.6<br>35.2 - 39.3	51.4 ± 8.3<br>40.2 - 83.3
Unknown	4	0	0	0	0	0
**T2D**						
N	140 (76%)	22 (96%)	18 (100%)	8 (100%)	9 (69%)	81 (67%)
Y	45 (24%)	1 (4.3%)	0 (0%)	0 (0%)	4 (31%)	40 (33%)
Unknown	3	0	0	0	0	1
**Glucose**<br>**[mmol/l]**	6.0 ± 3.0<br>2.2 - 22.1	5.0 ± 0.6<br>3.9 - 6.7	5.1 ± 1.4<br>2.2 - 7.7	4.8 ± 0.8<br>3.7 - 5.8	5.5 ± 3.1<br>2.9 - 13.0	6.4 ± 3.4<br>2.9 - 22.1
Unknown	49	7	5	3	4	30
**HbA1c**<br>**[%]**	6.0 ± 1.2<br>4.6 - 11.5	NA	5.4<br>5.4 - 5.4	5.2 ± 0.6<br>4.8 - 6.2	5.8 ± 1.5<br>4.6 - 9.3	6.1 ± 1.2<br>4.8 - 11.5
Unknown	89	23	17	4	5	40
**Cholesterol**<br>**[mmol/l]**	5.1 ± 1.0<br>1.4 - 8.0	5.6<br>5.6 - 5.6	4.6 ± 1.0<br>3.9 - 5.4	5.6 ± 0.6<br>4.9 - 6.2	5.4 ± 1.4<br>3.8 - 8.0	5.1 ± 1.0<br>1.4 - 7.4
Unknown	91	22	16	4	5	44
**LDL**<br>**[mmol/l]**	3.1 ± 0.8<br>1.3 - 5.3	3.5<br>3.5 3.5	2.6 ± 1.1<br>1.8 - 3.4	3.2 ± 0.6<br>2.6 - 3.9	2.8 ± 0.8<br>1.8 - 3.9	3.1 ± 0.9<br>1.3 - 5.3
Unknown	96	22	16	4	6	48
**HDL**<br>**[mmol/l]**	1.3 ± 0.4<br>0.6 - 2.4	1.5<br>1.5 1.5	1.4 ± 0.4<br>1.1 - 1.6	1.9 ± 0.6<br>1.2 - 2.4	1.3 ± 0.3<br>0.9 - 1.9	1.3 ± 0.3<br>0.6 - 2.3
Unknown	94	22	16	4	6	46
**Triglycerides**<br>**[mmol/l]**	1.9 ± 1.0<br>0.6 - 6.8	1.6<br>1.6 - 1.6	1.8 ± 1.5<br>0.7 - 2.9	1.5 ± 1.0<br>0.8 - 3.1	2.7 ± 1.8<br>1.1 - 5.7	1.8 ± 0.9<br>0.6 - 6.8
Unknown	92	22	16	4	5	45

Continuous variables are specified as mean ± SD with the second row specifying minimal and maximal values. Categorical variables are given as count with percentages specified in brackets

Table S2: Pearson correlations between mean adipocyte size and anthropometry/laboratory chemistry. Associations with a significance level of $p < 0.05$ are written in bold. Associations that remained significant after Bonferroni correction for multiple testing are written in bold and cursive.

	Dep.	**BMI** [kg/m^2] n_{sc} = 158 n_{vc} =184		**Age** [years] n_{sc} = 161 n_{vc} = 188		**Glucose** [mmol/l] n_{sc} = 120 n_{vc} = 139		**HbA$_{1C}$** [%] n_{sc} = 90 n_{vc} = 99		**Cholesterol** [mmol/l] n_{sc} = 89 n_{vc} = 97		**LDL** [mmol/l] n_{sc} = 83 n_{vc} = 92		**HDL** [mmol/l] n_{sc} = 86 n_{vc} = 94		**Triglycerides** [mmol/l] n_{sc} = 88 n_{vc} = 96	
		$r_{pearson}$	p	$r_{pearson}$	p	$r_{pearson}$	p	$r_{pearson}$	p	$r_{pearson}$	p	$r_{pearson}$	p	$r_{pearson}$	p	$r_{pearson}$	p
Mean area [µm^2]	sc	0.49	***5E-11***	-0.19	**0.015**	0.12	0.193	0.07	0.529	-0.20	0.055	-0.11	0.313	-0.01	9.2E-01	0.11	0.328
	vc	0.47	***2E-11***	-0.02	0.762	0.26	***0.002***	0.32	***0.001***	-0.10	0.345	0.04	0.706	-0.34	***7.4E-04***	0.20	**0.050**
Mean diameter [µm]	sc	0.49	***6E-11***	-0.18	**0.024**	0.11	0.232	0.06	0.575	-0.22	**0.041**	-0.12	0.281	0.00	1.000	0.09	0.420
	vc	0.48	***9E-12***	-0.01	0.921	0.26	***0.002***	0.31	***0.002***	-0.06	0.542	0.03	0.783	-0.32	***1.6E-03***	0.19	0.063
Mean volume [pl]	sc	0.48	***1E-10***	-0.20	**0.013**	0.12	0.181	0.07	0.517	-0.19	0.072	-0.10	0.357	-0.02	0.872	0.12	0.280
	vc	0.46	***6E-11***	-0.03	0.677	0.27	***0.002***	0.31	***0.002***	-0.13	0.221	0.03	0.783	-0.35	***5.4E-04***	0.20	0.051
Mean surf. area [µm^2]	sc	0.49	***5E-11***	-0.19	**0.015**	0.12	0.193	0.07	0.529	-0.20	0.055	-0.11	0.313	-0.01	0.917	0.11	0.328
	vc	0.47	***2E-11***	-0.02	0.762	0.26	***0.002***	0.32	***0.001***	-0.10	0.345	0.02	0.831	-0.34	***7.4E-04***	0.20	**0.050**

Table S3: Multiple linear regression analysis on mean adipocyte diameter and anthropometry/laboratory values. Mean adipocyte diameter from histology was set as the dependent variable. To exclude an influence of the BMI on the correlation between mean adipocyte size and the independent variable BMI was used as a second independent variable to adjust for in multiple linear regression. p-values < 0.05 are written in bold.

**Variable**	Depot (n)	ß adj. for BMI	p adj. for BMI	Adj. R^2 Model
Age	sc (n = 158)	-0.02	0.644	0.23
	vc (n = 184)	0.01	**0.028**	0.24
Sex (male)	sc (n = 158)	-0.98	0.426	0.23
	vc (n = 184)	3.34	**0.006**	0.25
T2D	sc (n = 158)	0.56	0.666	0.23
	vc (n = 183)	3.34	**0.010**	0.25
Glucose [mmol/l]	sc (n = 117)	0.16	0.443	0.22
	vc (n = 135)	0.56	**0.004**	0.28
HbA1C [%]	sc (n = 87)	0.18	0.775	0.06
	vc (n = 95)	1.59	**0.002**	0.15
Cholesterol [mmol/l]	sc (n = 86)	- 1.22	0.084	0.11
	vc (n = 93)	- 0.19	0.769	0.10
LDL [mmol/l]	sc (n = 80)	- 0.82	0.353	0.09
	vc (n = 88)	0.17	0.834	0.12
HDL [mmol/l]	sc (n = 83)	1.60	0.446	0.08
	vc (n = 90)	-4.55	**0.015**	0.14
Triglycerides [mmol/l]	sc (n = 85)	0.60	0.392	0.09
	vc (n = 92)	1.58	**0.017**	0.15

Table S4: Correlations between mean adipocyte diameter and mitochondrial respiratory capacity of mature adipocytes. Dependent on the distribution of the data either pearson or spearman correlations were used to assess the relationship between mean adipocyte diameter and respiratory chain function. Significant correlations are written in bold.

**Respiratory state**	**Depot**	**Mean Diameter**		
		Pearson/Spearman Correlation coefficient	p-value	Method
Free Oxphos capacity (pmol/(s*ng DNA))	sc (24)	- 0.41	**0.045**	Pearson
	vc (35)	- 0.21	0.209	Pearson
Oxphos capacity (pmol/(s*ng DNA))	sc (24)	- 0.53	**0.008**	Pearson
	vc (35)	- 0.36	**0.035**	Spearman
Leak respiration oligomycin (pmol/(s*ng DNA))	sc (24)	- 0.40	**0.050**	Pearson
	vc (35)	- 0.37	**0.026**	Pearson
Electron transfer system capacity (pmol/(s*ng DNA))	sc (24)	- 0.59	**0.003**	Pearson
	vc (35)	- 0.33	0.058	Pearson

Table S5: Multiple linear regression analysis on mean adipocyte diameter and adipocyte respiratory chain function. Mean adipocyte diameter from histology was set as the dependent variable. To exclude an influence of the BMI on the correlation between mean adipocyte size and mitochondrial respiratory capacity BMI was used as a second independent variable to adjust for in multiple linear regression. p-values < 0.05 are written in bold.

**Dependent variable**	**Depot (n)**	**ß adj. for BMI**	**p adj. for BMI**	**Adj. R^2 Model**
Free Oxphos capacity (pmol/(s*ng DNA))	sc (24)	-0.05	0.630	0.31
	vc (35)	-0.07	0.273	0.11
Oxphos capacity (pmol/(s*ng DNA))	sc (24)	-0.09	0.254	0.34
	vc (35)	0.17	0.126	0.14
Leak respiration oligomycin (pmol/(s*ng DNA))	sc (24)	-0.16	0.227	0.35
	vc (35)	-0.28	0.053	0.18
Electron transfer system capacity (pmol/(s*ng DNA))	sc (24)	-0.14	0.131	0.37
	vc (35)	-0.11	0.083	0.14

Table S6: Correlations between mean adipocyte diameter and mitochondrial respiratory capacity of mature adipocytes with 200 sampled cells to determine mean adipocyte diameter. Dependent on the distribution of the data either pearson or spearman correlations were used to assess the relationship between mean adipocyte diameter and respiratory chain function. Significant correlations are written in bold.

**Respiratory state**	**Depot**	**Mean Diameter**		
		Pearson/Spearman Correlation coefficient	p-value	Method
Free Oxphos capacity (pmol/(s*ng DNA))	sc (32)	-0.45	**0.010**	Pearson
	vc (41)	-0.21	0.182	Pearson
Oxphos capacity (pmol/(s*ng DNA))	sc (32)	-0.59	**3.83E-04**	Pearson
	vc (41)	-0.33	**0.033**	Spearman
Leak respiration oligomycin (pmol/(s*ng DNA))	sc (32)	-0.50	**0.003**	Pearson
	vc (41)	-0.32	**0.037**	Pearson
Electron transfer system capacity (pmol/(s*ng DNA))	sc (32)	-0.67	**3.18E-05**	Pearson
	vc (41)	-0.34	**0.031**	Pearson

Table S7: Multiple linear regression analysis on mean adipocyte diameter and adipocyte respiratory chain function with 200 sampled cells to determine mean adipocyte diameter. Mean adipocyte diameter from histology was set as the dependent variable. To exclude an influence of the BMI on the correlation between mean adipocyte size and mitochondrial respiratory capacity BMI was used as a second independent variable to adjust for in multiple linear regression. p-values < 0.05 are written in bold.

Variable	Depot (n)	ß adj. for BMI	p adj. for BMI	Adj. R^2 Model
Free Oxphos capacity (pmol/(s*ng DNA))	sc (32)	-0.13	0.101	0.25
	vc (41)	-0.08	0.255	0.08
Oxphos capacity (pmol/(s*ng DNA))	sc (32)	-0.16	**0.007**	0.36
	vc (41)	-0.09	0.104	0.10
Leak respiration oligomycin (pmol/(s*ng DNA))	sc (32)	-0.29	**0.011**	0.34
	vc (41)	-0.29	0.057	0.13
Electron transfer system capacity (pmol/(s*ng DNA))	sc (32)	-0.221	**0.001**	0.44
	vc (41)	-0.14	**0.045**	0.12

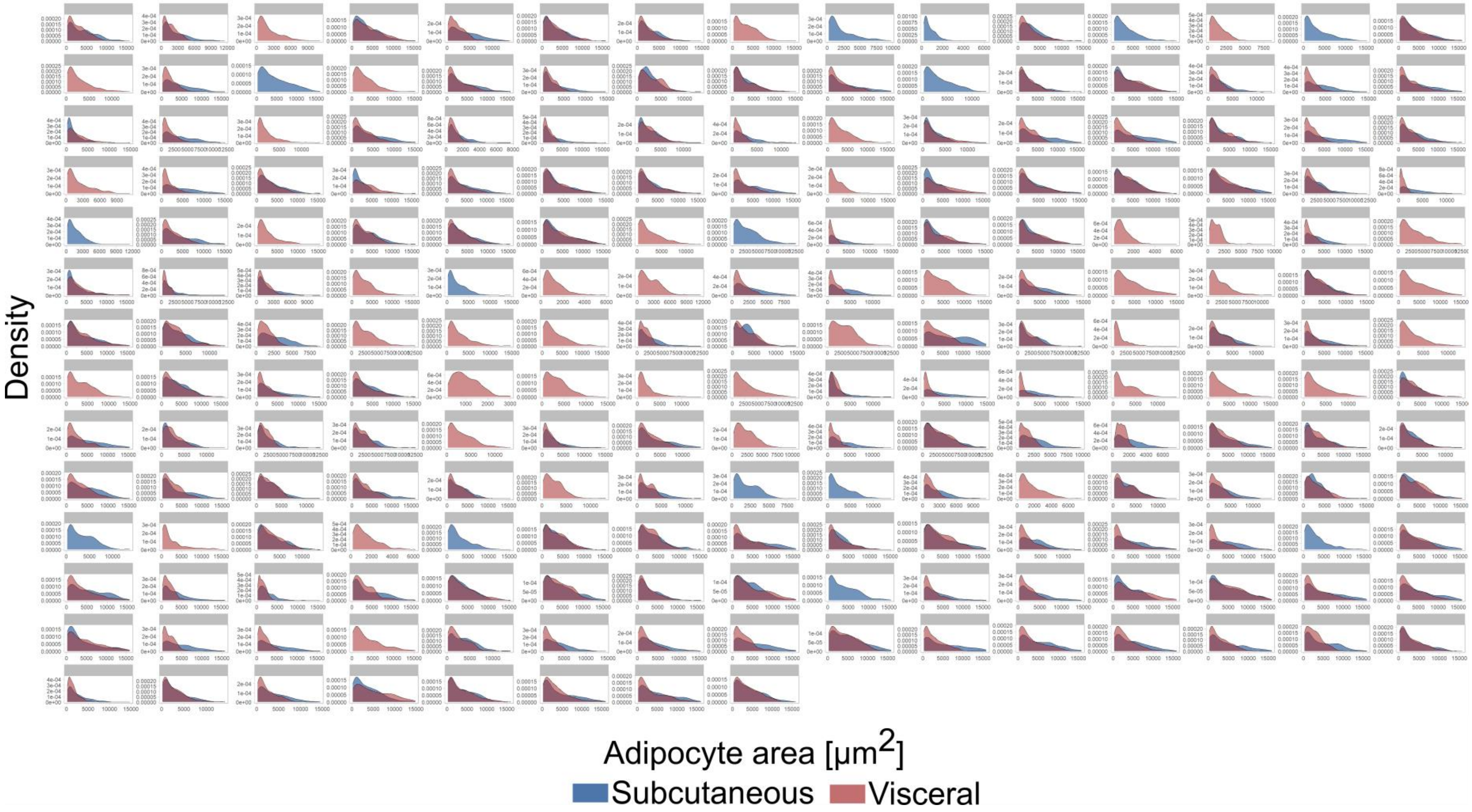

Figure S1: Individual density plots for sc and vc adipocyte area based on 500 sampled cells

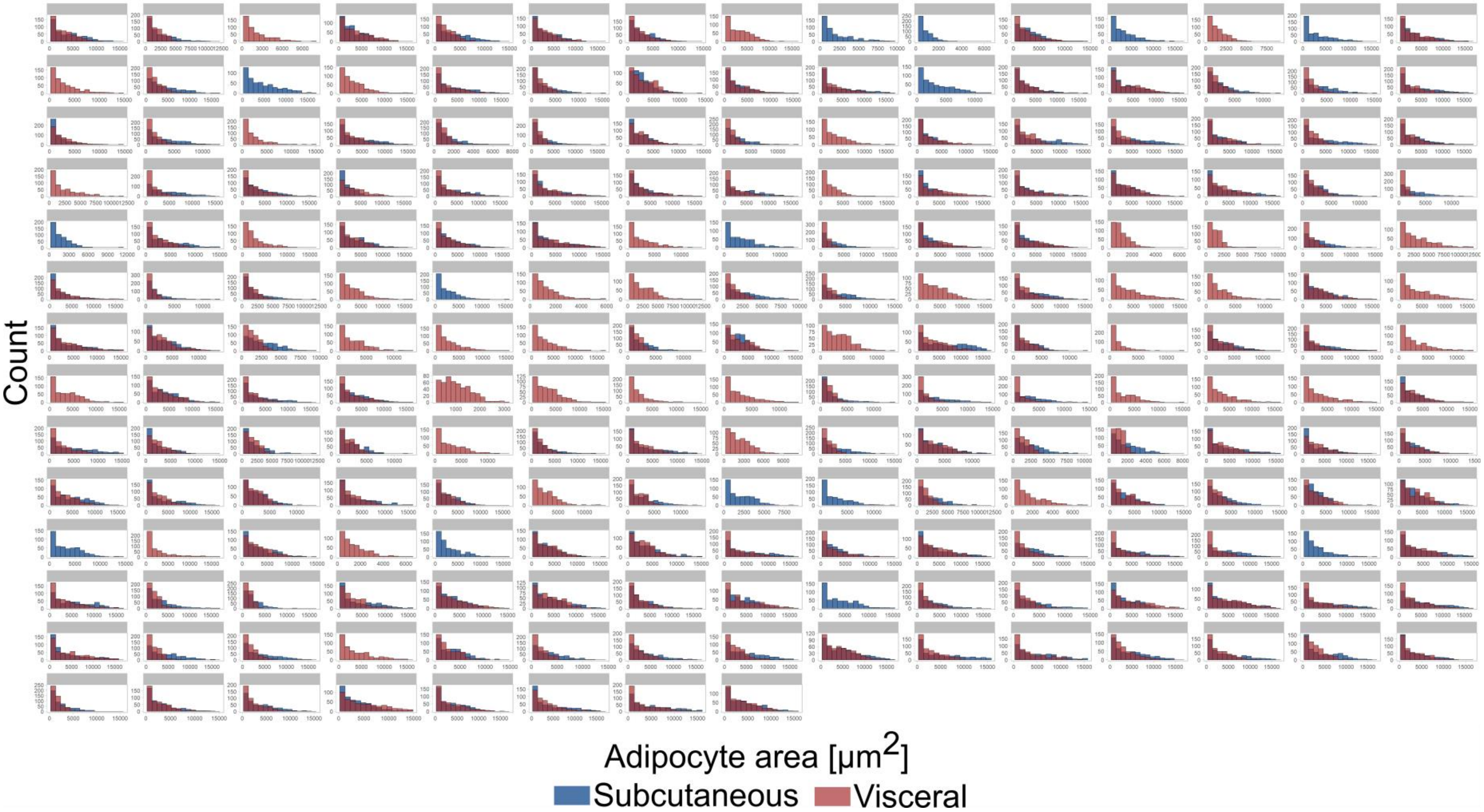

Figure S2: Individual histograms for sc and vc adipocyte area based on 500 sampled cells

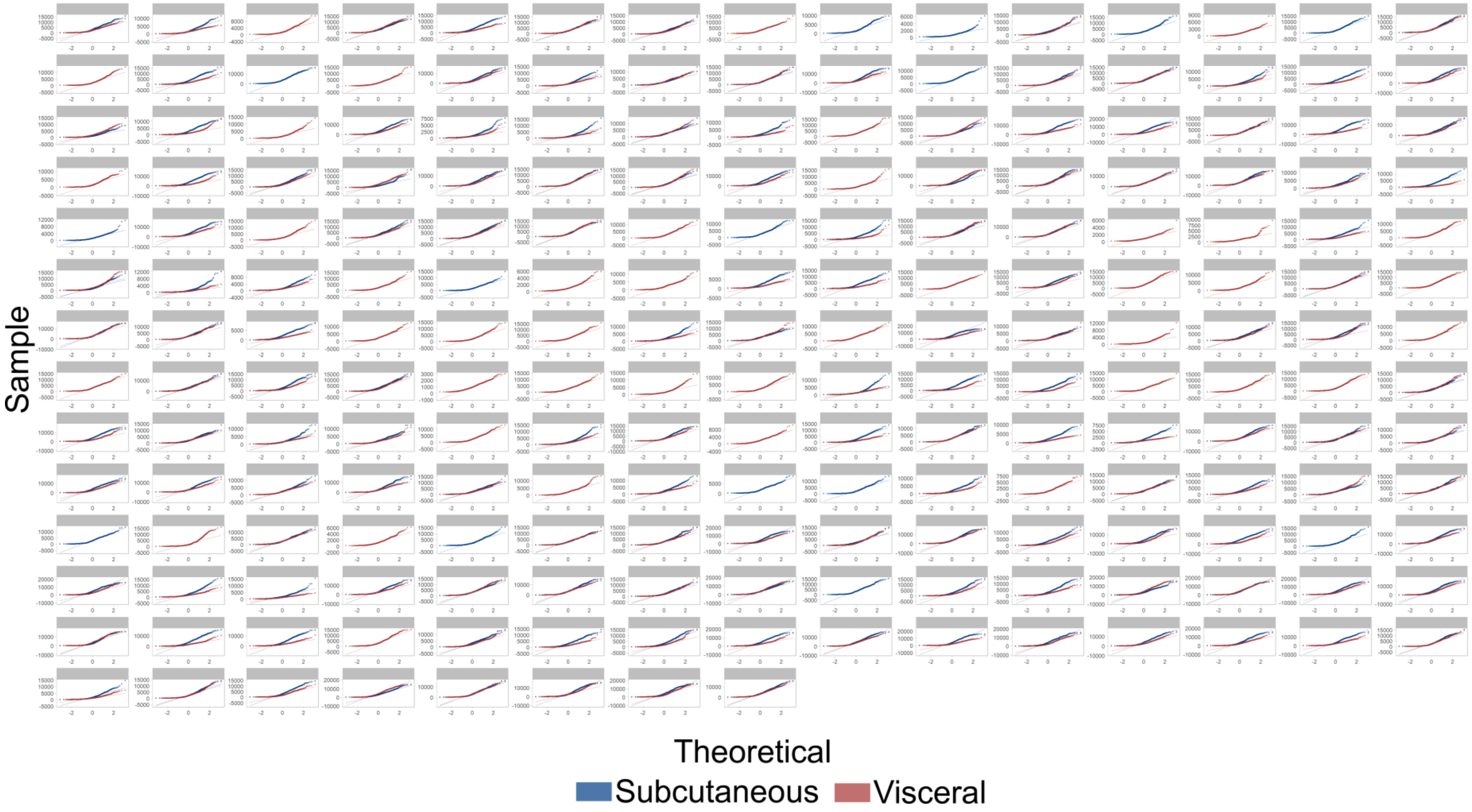

Figure S3: Individual QQ plots for sc and vc adipocyte area based on 500 sampled cells

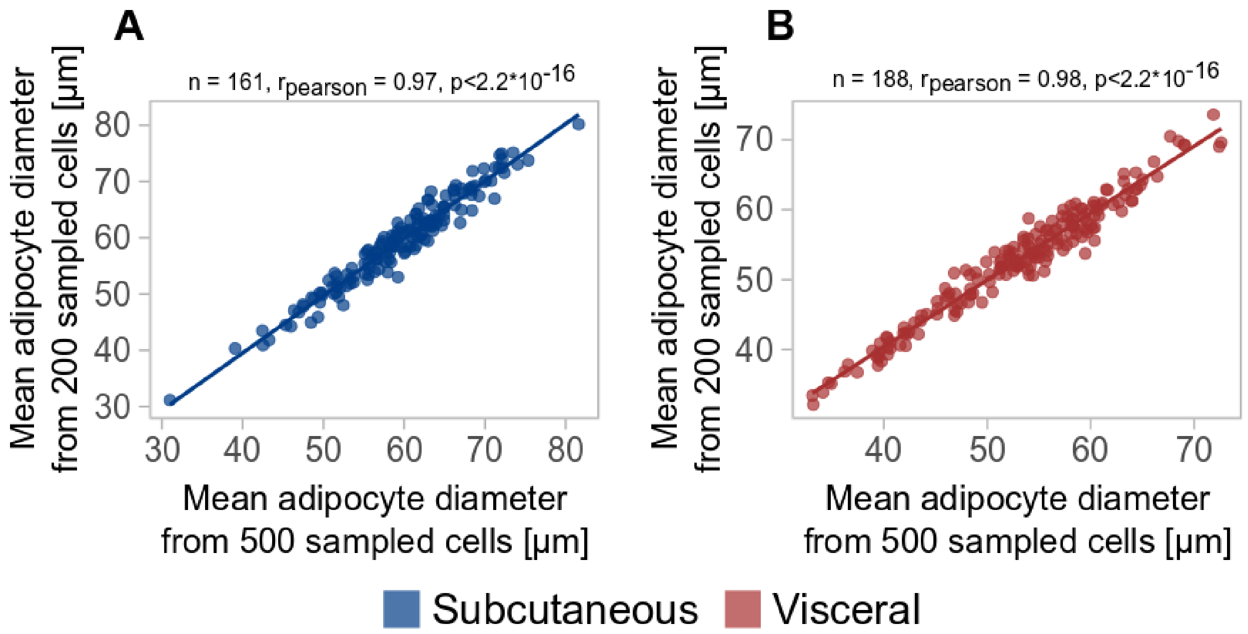

Figure S4: Correlations between sc (A) and vc (B) mean adipocyte diameter from 500 and 200 sampled cells.

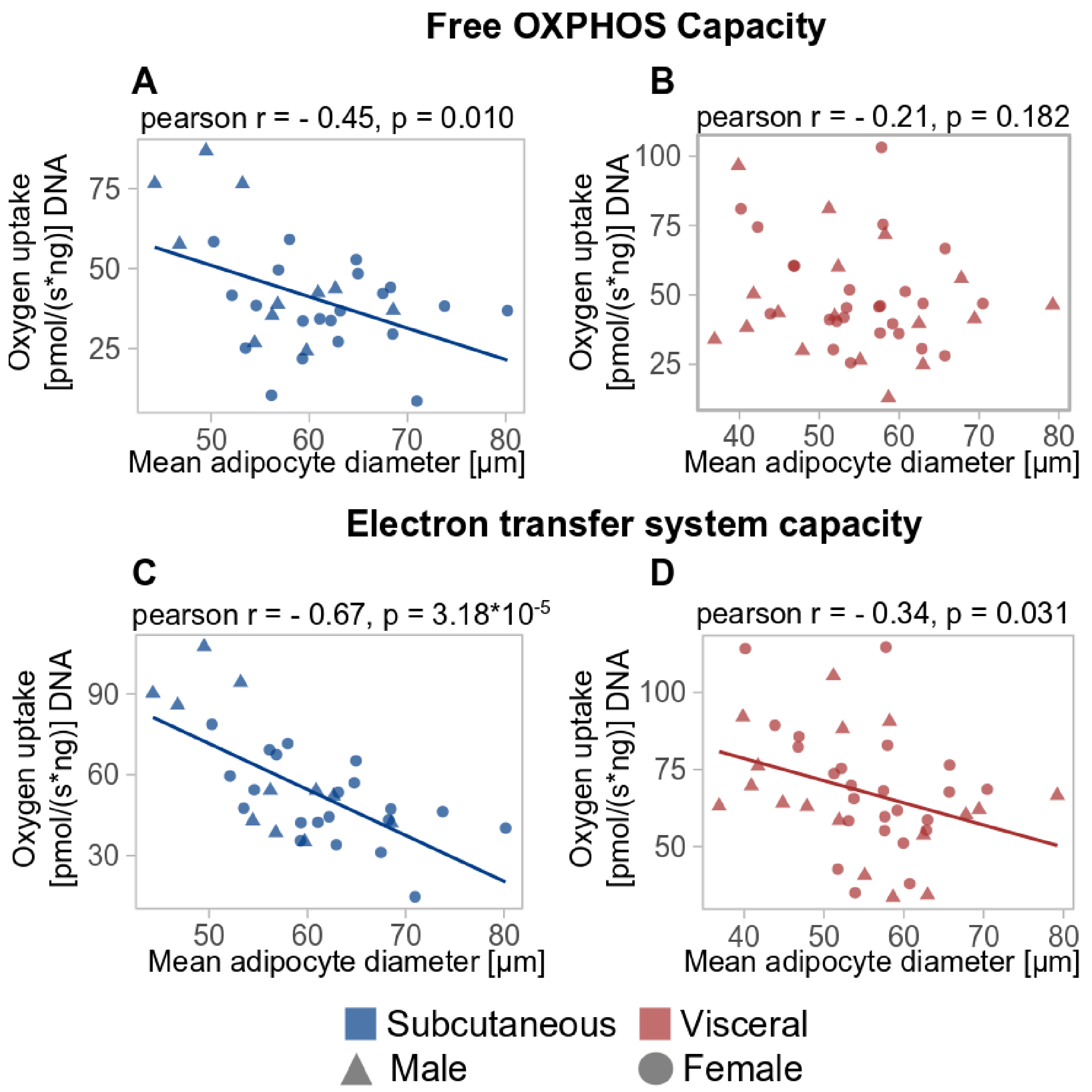

Figure S5: Correlations between mean adipocyte diameter and mitochondrial respiratory capacity of mature adipocytes with 200 sampled cells to determine mean adipocyte diameter.

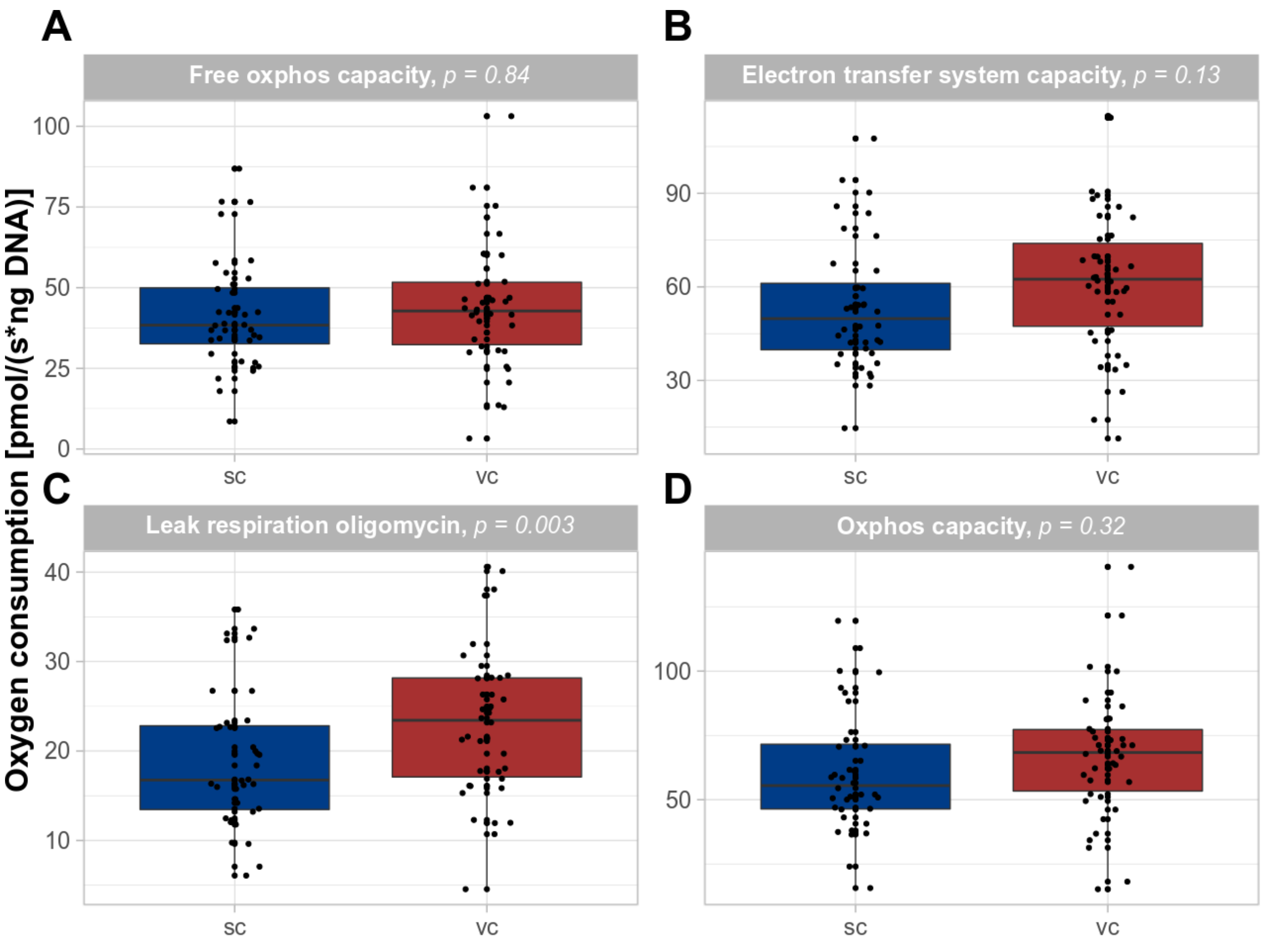

Figure S6: Differences in respiratory states according to adipose tissue depot. Paired sample t-tests were used to test for differences in respiration in 51 samples. No significant differences were found except for leak respiration in the presence of oligomycin (p = 0.003). P-values originate from two-sided paired t-tests.
